# A huge asymptomatic pheochromocytoma

**DOI:** 10.1002/ccr3.1566

**Published:** 2018-05-08

**Authors:** Nikolaos Machairas, Dimetrios Papaconstantinou, Anna Papala, Argyrios Ioannidis, Paul Patapis, Evangelos P. Misiakos

**Affiliations:** ^1^ 3rd Department of Surgery National and Kapodistrian University of Athens Medical School Athens Greece

**Keywords:** cystic tumor, huge, pheochromocytoma, retroperitoneal

## Abstract

Due to their evolution in the retroperitoneal space, pheochromocytomas may grow significantly in size and remain asymptomatic for a long period of time. Normal values of urine catecholamine levels must not preclude the diagnosis of these endocrine lesions.

## 1 CASE PRESENTATION

**Figure 1:**
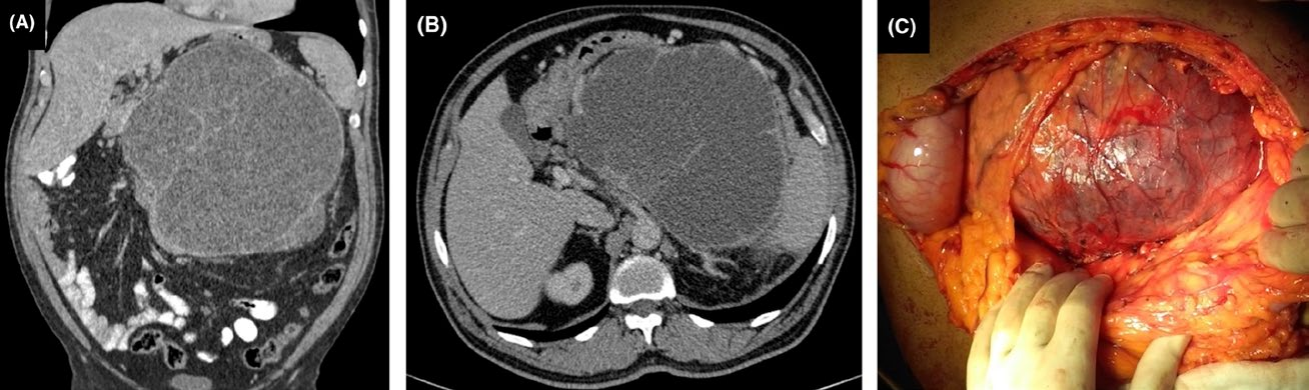
A, CT imaging of the retroperitoneal cystic lesion (coronal view). B, CT imaging of the retroperitoneal cystic lesion (transverse view). C, Intraoperative view of the lesion

A 55‐year‐old male patient presented to our department with progressively worsening, right hypochondrium pain. He complained for abdominal distention and early satiety for the last 2 months. His history was significant for a one‐year onset of mildly elevated arterial pressure, successfully managed with administration of low‐dose amlodipine. Clinical examination revealed a voluminous palpable mass occupying the left quadrat of his abdomen. Abdominal ultrasound imaging showed a huge cystic mass posterior to the pancreas, compressing the stomach. Abdominal computer tomography (CT) confirmed the presence of a huge cystic retroperitoneal lesion, which measured 22 × 22 × 10 cm (Figure 1A,B). The lesion showed close intimacy to the posterior aspect of the pancreas. Endoscopic ultrasound fluid aspiration was negative for CA19‐9 and amylase, whereas biopsy samples were inconclusive on the nature of the lesion. Albeit the fact that the lesion was not shown to arise from the left adrenal, 24‐hour urine catecholamine levels were also examined and were within normal range. The patient's vital signs and blood CEA and CA19‐9 were within normal range. The patient underwent complete excision of the cystic mass. Histology demonstrated the presence of pheochromocytoma, which arose from adrenal tissue and demonstrated immunohistopositivity to chromogranin and synaptophysin. Due to lesion size and microvascular invasion, it was categorized as malignant.

Due to their evolution in the retroperitoneal space, pheochromocytomas may grow significantly in size and remain asymptomatic for a long period of time.^1^ Moreover, contrary to solid, cystic pheochromocytomas may not present typical clinical symptomatology or urine values of catecholamine metabolites may be found within normal range.^2^ Such atypical presentation makes the preoperative diagnosis of giant retroperitoneal cystic lesions challenging.

## CONFLICT OF INTEREST

None declared.

## AUTHORSHIP

NM: designed and conceived the study, acquired the data, wrote the manuscript. DP, AP, and AI: acquired the data, wrote the manuscript. PP and EPM: analyzed and interpreted the data.
